# Apparent Diffusion Coefficient in Invasive Ductal Breast Carcinoma: Correlation with Detailed Histologic Features and the Enhancement Ratio on Dynamic Contrast-Enhanced MR Images

**DOI:** 10.1155/2010/821048

**Published:** 2010-09-02

**Authors:** Roka Namoto Matsubayashi, Teruhiko Fujii, Kotaro Yasumori, Toru Muranaka, Seiya Momosaki

**Affiliations:** ^1^Department of Radiology, National Hospital Organization Kyushu Medical Center, 1-8-1 Jigyohama, Chuo-ku, Fukuoka 810-8563, Japan; ^2^Breast Care Center, National Hospital Organization Kyushu Medical Center, 1-8-1 Jigyohama, Chuo-ku, Fukuoka 810-8563, Japan; ^3^Department of Surgery, National Hospital Organization Kyushu Medical Center, 1-8-1 Jigyohama, Chuo-ku, Fukuoka 810-8563, Japan; ^4^Clinical Research Institute, National Hospital Organization Kyushu Medical Center, 1-8-1 Jigyohama, Chuo-ku, Fukuoka 810-8563, Japan; ^5^Department of Pathology, National Hospital Organization Kyushu Medical Center, 1-8-1 Jigyohama, Chuo-ku, Fukuoka 810-8563, Japan

## Abstract

*Purpose*. To investigate the correlation of Apperent Diffusion Coefficient (ADC) values in invasive ductal breast carcinomas with detailed histologic features and enhancement ratios on dynamic contrast-enhanced MRI. *Methods and Materials*. Dynamic MR images and diffusion-weighted images (DWIs) of invasive ductal breast carcinomas were reviewed in 25 (26 lesions) women. In each patient, DWI, T2WI, T1WI, and dynamic images were obtained. The ADC values of the 26 carcinomas were calculated with b-factors of 0 and 1000 s/mm^2^ using echoplanar DWI. Correlations of the ADC values were examined on dynamic MRI with enhancement ratios (early to delayed phase: E/D ratio) and detailed histologic findings for each lesion, including cellular density, the size of cancer nests, and architectural features of the stroma (broad, narrow, and delicate) between cancer nests. *Results*. The mean ADC was 0.915 ± 0.151 × 10^−3^ mm^2^/sec. Cellular density was significantly correlated with ADC values (*P* = .0184) and E/D ratios (*P* = .0315). The ADC values were also significantly correlated to features of the stroma (broad to narrow, *P* = .0366). *Conclusion*. The findings suggest that DWIs reflect the growth patterns of carcinomas, including cellular density and architectural features of the stroma, and E/D ratios may also be closely correlated to cellular density.

## 1. Introduction

Diffusion-weighted (DW) imaging has become an important method for diagnosis of breast lesions. Recent studies [1–5] have shown the usefulness of DW imaging for detecting breast tumors and distinguishing between malignant and benign breast lesions. In many tumors, histologic differentiation reflects the cellular density, and several studies [6–9] have demonstrated a relationship between apparent diffusion coefficient (ADC) values and tumor cellular density. 

Typically, invasive ductal carcinoma shows early strong enhancement due to the expression of many angiogenic or growth factors, including vascular endothelial growth factor (VEGF) and tumor neovascularity. Pathologically, invasive ductal breast carcinomas are comprised of cancer nests, stromas between these nests, and some ductal components. We have shown that histologic morphologic features such as the size of the cancer nests, the width of the stroma, fibrosis, angiogenesis, and patterns of VEGF expression affect enhancement patterns of breast carcinomas [10]. Additionally, these histologic parameters are closely related with growth patterns of breast carcinomas. 

The aim of the study was to investigate the histologic features that affect the ADC in invasive ductal breast carcinomas. To our knowledge, correlations of ADC values with enhancement patterns on dynamic contrast-enhanced MR images and morphologic histologic features in invasive ductal breast carcinomas have not been reported previously. 

## 2. Material and Methods

### 2.1. Patients

Twenty-five consecutive women (26 lesions) (age 30–74 years old, mean 59.2 ± 10.9 years old) with invasive ductal breast carcinoma who underwent partial or total mastectomy after MR imaging at our institution between September 2005 and August 2006 were selected for examination. The lesion was initially detected by physical examination, mammography, or ultrasonography. None of the patients had undergone chemotherapy or large-core needle biopsy for tissue sampling before the MR examination.

### 2.2. MR Imaging

MR imaging was performed using a 1.5T whole-body imager (Magnetom Symphony; Siemens AG, Erlangen, Germany). The affected side in each patient was examined using a dedicated breast coil with the patient in the prone position. DW images were acquired using a multisection single-shot short tau inversion recovery (STIR) echoplanar sequence in the transverse or sagittal plane. Following DW imaging, fat-suppressed T2- and T1-weighted and dynamic images were obtained. Subtraction images were produced from dynamic images for identification of enhancement. The imaging parameters were as follows: DWI (TR/TE/TI = 5400/80/180 msec, matrix = 50 × 128, slice thickness (SL) = 5 mm, FOV = 131 × 300 mm, b factor = 0 and 1000 s/mm^2^, with a motion probing gradient (MPG) applied along the *X*, *Y*, and *Z* axes), 2D fat-suppressed T2-weighted turbo spin-echo pulse sequence (TR/TE = 4500/84 msec, matrix = 460 × 512, SL = 5 mm, field of view (FOV) = 200 × 200 mm), and a three-dimensional fat-suppressed T1-weighted FLASH pulse sequence (TR/TE = 5.9/2.4 msec, matrix = 141 × 256, SL = 1.5 mm, FOV = 150 × 220 mm). The latter sequence was performed before and during intravenous contrast enhancement with 0.1 mmol gadopentetate dimeglumine (Magnevist; Schering, Berlin, Germany) per kilogram of body weight. A bolus of contrast agent was injected intravenously using a dedicated infusion pump at a rate of 1 ml/s, followed by a 20-ml saline solution flush. Sequential multisection, whole-breast images were obtained in the sagittal plane at 30-second intervals for 5 minutes. In all patients, late sagittal T1-weighted (TR/TE = 420/10 msec, matrix = 384 × 512, SL = 5 mm, FOV = 200 × 200 mm) and transverse T1-weighted (TR/TE = 420/10 msec, matrix = 384 × 512, SL = 5 mm, FOV = 131 × 300 mm) images were obtained for the bilateral breasts.

### 2.3. Image Analysis

**Interpretation of MR Images**

Two of the authors (R. N. Matsubayashi and T. Fujii) interpreted the MR images. We recorded the size, shape, and enhancement pattern of each lesion but did not use the ACR MRI BI-RADS classification.

**Measurement of the ADC**

**Figure 1:**
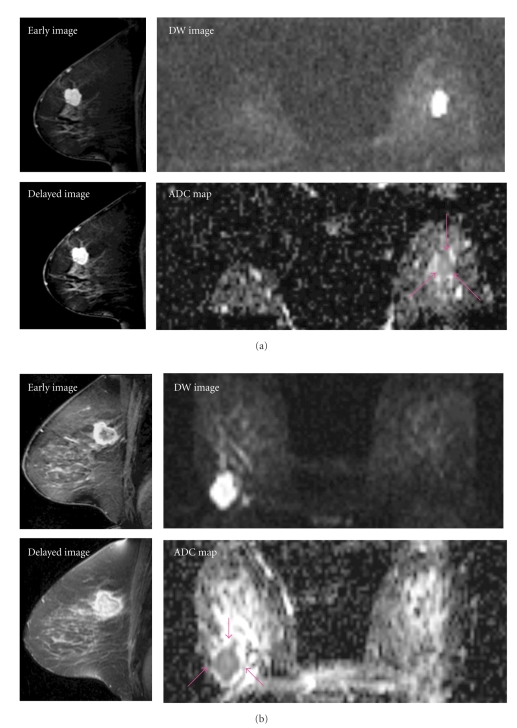
MR images of representative cases. (a) Case 3: a 59-year-old woman with invasive carcinoma of the left breast. E/D ratio: 1.03, ADC value 0.850 × 10^−3^ mm^2^/s. A well-demarcated mass with strong enhancement is observed. The mass shows high intensity on DWI (b = 1000). (b) Case 13: a 72-year-old woman with invasive carcinoma in the right breast. E/D ratio: 0.90, ADC value 0.975 × 10^−3^ mm^2^/s. A mass with early rim enhancement with delayed internal enhancement is shown. The mass shows high intensity on DWI (b = 1000). The central portion shows relatively low signal intensity than that in the periphery. The central area showing delayed enhancement may have abundant fibrous stroma.

Based on the DW images, we measured the ADC values of 26 carcinomas. ADC values were calculated with b-factors of 0 and 1000 s/mm^2^ using echoplanar DW images. A region of interest (ROI) of as large a size as possible was positioned over the tumor to avoid necrosis or scar (nonenhanced area) and artifacts, based on dynamic MR images (Figures 1(a) and 1(b)). Positioning of the ROI of each lesion was performed by a radiologist (R. N. Matsubayashi).

When the lesions were ring-like on DW images, we positioned the ROI in the peripheral portion. The signal intensities in the ROI corresponded to the two different b values.

**Enhancement Ratio on MR Images**

The enhancement ratio on dynamic contrast-enhanced MR images in the early (60 sec) to the delayed (300 sec) phase (E/D ratio) was calculated for each lesion. When the lesion showed rim enhancement, the E/D ratio was calculated at the peripheral region.

### 2.4. Histopathologic Analysis

 Two of the authors (R. N. Matsubayashi: who has degree of pathology and S. Momosaki: general pathologist) performed the histopathologic analysis.

**Figure 2:**
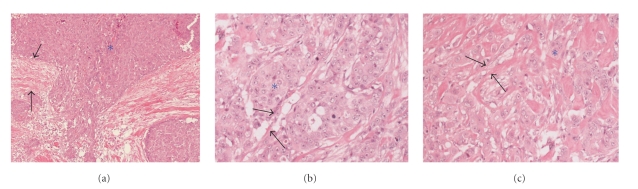
Cancer nests at invasive sites are classified based on the mean length of the major axis: small, medium, or large. Features of the stroma between the nests are classified as delicate, narrow, or broad (nests: asterisk, stroma: black arrows). (a) Nests: large, stroma: broad. (b) Nests: medium, stroma: delicate. (c) Nests: small, stroma: narrow. All sections are hematoxylin-eosin stained.

Evaluation of morphologic features was performed using slices stained with hematoxylin-eosin. Cellular density, the size of the cancer nests, and the architectural features of the stroma between the cancer nests were examined (Figure 2). To determine the cellular density, the number of nuclei of cancer cells in 10 high-power fields (x400) for each lesion was counted, and the mean value was recorded. To assess the size of cancer nests and architectural features of the stroma, the major axis of 10 cancer nests and the width of the stroma at 10 points were also measured for each lesion. Based on the mean length of the major axis, the sizes of the cancer nests were determined and classified as small (<40 *μ*m), medium (40–100 *μ*m), or large (>100 *μ*m). The mean width and morphology of the stroma between the cancer nests were determined, and the stroma was classified as delicate (<10 *μ*m), narrow (10–50 *μ*m), or broad (>50 *μ*m).

### 2.5. Statistical Analysis

Statistical comparisons were performed using Fisher's protected least significant difference test or Spearman's log rank test. The correlation of ADC values with the E/D ratio on dynamic contrast-enhanced MR images was examined. ADC values were also compared between groups, for which the status of each histologic parameter, size of cancer nests, and stroma features were determined. A *P*-value of less than  .05 was considered to be statistically significant. Data are expressed as means ± SD. Statistical analysis was performed using StatView software.

## 3. Results

**Figure 3:**
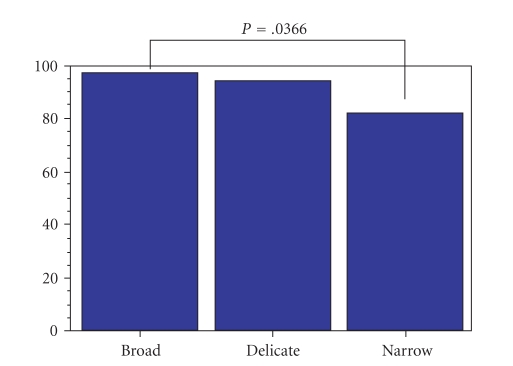
ADC values were significantly correlated with stroma features (broad to narrow, *P* = .0366). D: delicate, N: narrow, B: broad. ADC:×10^−3^ mm^2^/s. S: small, M: medium, L: large, D: delicate, N: narrow, B: broad, E/D: enhancement ratio of early/delayed phase.

Clinical data, MR imaging and histologic features, ADCs, and the E/D ratio on contrast-enhanced MR images for all patients are summarized in Table 1. The sizes of lesions were 0.8–3.5 cm (mean 1.9 ± 0.8 cm). The mean ADC value of the lesions was 0.915 ± 0.151 × 10^−3^ mm^2^/s, and the mean enhancement ratio was 0.962 ± 0.1. Cellular densities were significantly correlated with ADC values (*P* = .0184) and E/D ratios (*P* = .0315). ADC values were also significantly correlated with stroma features (broad to narrow, *P* = .0366) (Figure 3). There was no correlation between ADC values and E/D ratios and the size of cancer nests.

## 4. Discussion

MR imaging is useful for diagnosis of breast carcinomas [11] and to assist in selection of appropriate treatment. Due to overexpression of growth factors, breast carcinomas show high angiogenesis, especially at invasive sites. The expression level of one of the angiogenic factors, VEGF, correlates with the histologic grade and a variety of clinical and prognostic factors [12–15]. Dynamic contrast-enhanced MR imaging has been used to evaluate the vascular supply to breast carcinomas. Invasive ductal breast carcinomas show strong early enhancement with prolonged or reduced delayed enhancement in such imaging, and these enhancement patterns are closely correlated with histologic morphologic features [10]. 

DWI is a useful tool for diagnosis of cerebral infarcts and other intracranial lesions and has recently been used for diagnosis and detection of neoplasms in several organs. In many carcinomas, cellular density is higher in lesions of higher grade, and DWI may reflect the carcinoma growth pattern. DWI has also been used for body imaging, since it allows clear detection of breast carcinomas and is helpful in determination of the extent of the carcinoma and in the differentiation between malignant and benign lesions [1–5]. DWI is also used to calculate ADC values, which can reflect cellular density; thus, this technique provides a lot of useful information for treatment decisions. 

Kuroki et al. [3] showed that diffusion in breast carcinoma is significantly lower than that in benign masses, and a significant difference in diffusion has been seen between invasive ductal carcinoma and pure or predominant non-invasive ductal carcinoma. Several reports have also shown a correlation between the stage of liver fibrosis and ADCs [16, 17]. Since breast carcinoma often has fibrotic stroma, we hypothesized that the degree of fibrosis might also be reflected by the ADC.

Both cellular density and histologic architectural variation of cancer nests and stroma may be affected by the growth pattern, including the histologic grade of the individual breast carcinoma. In the present study, enhancement ratios of breast carcinomas on dynamic MR images closely correlated with cellular density and the histologic, morphologic features. These results suggest that both DW imaging and dynamic studies can provide detailed histological or biological information about the lesions. Prediction of growth patterns of tumors from imaging findings may be useful for planning treatment for breast carcinoma, and we suggest that a combination of these imaging techniques may become an important tool to evaluate the effectiveness of chemotherapy or irradiation for breast carcinoma. 

The study is limited by the relatively small number of patients. Also, it was difficult to evaluate ADCs of tiny lesions on our DW images because the images were distorted in the breast periphery. Parallel imaging techniques including SENSE may be able to reduce these artifacts (including motion artifacts) and shorten the scanning time. Additionally, for the optimal measurement of ADC values, patients with prior large-core needle biopsy must be excluded because the blood may affect the ADC. And then, we consider that the ROI must cover the enhancing area as large as possible avoiding necrosis or scar to correctly reflect the cellular density of the tumor. 

## 5. Conclusion

Despite the small number of cases, our findings suggest that enhancement ratios on dynamic MR imaging reflect the growth patterns of invasive ductal breast carcinomas, including the cellular density. The cellular density and cancer stroma also appear to be closely correlated with ADC values. Both DW imaging and dynamic studies provide detailed information about invasive breast carcinomas, and our results demonstrate the potential for use of DWI in the assessment of treatment for invasive ductal carcinoma. 

## Figures and Tables

**Table 1:** Clinical data, MR imaging and histologic features, ADCs, and the E/D ratio on contrast-enhanced MR images.

Case Number	Age	Tumor diameter (cm)	Cellular density	Size of nest	Type of stroma	E/D	ADC
1	61	1.4 × 1.0	1038	M	N	0.98	0.564
2	69	3.0 × 2.3	315	M	N	0.96	0.830
3	59	1.6 × 0.9	527	L	D	1.03	0.850
4	62	0.8 × 0.7	301	S	B	1.02	0.940
5	60	2.8 × 2.6	324	S	B	0.88	0.910
6	49	0.8 × 0.8	902	M	D	1.10	0.863
7	30	1.5 × 1.3	428	S	B	0.91	0.885
8	67	3.5 × 3.4	502	L	B	0.92	0.718
9	58	2.0 × 1.6	766	M	D	1.06	0.971
10	65	2.5 × 1.9	278	S	B	0.84	1.118
11	45	1.7 × 1.3	336	M	N	0.99	0.930
12	58	1.0 × 0.8	303	M	N	0.96	0.858
13	72	2.3 × 2.0	211	M	D	0.90	0.975
14	61	2.5 × 1.9	224	L	N	0.76	1.218
15	67	2.0 × 1.7	504	L	D	1.11	0.828
16	41	1.9 × 1.7	508	S	N	0.92	0.837
17	73	0.9 × 0.8	856	M	D	0.93	0.866
18	68	1.8 × 1.2	886	L	B	0.99	0.874
19	69	2.5 × 1.7	566	L	D	1.08	0.934
20	63	3.2 × 2.9	452	S	B	0.84	0.865
21	63	2.2 × 1.5	621	S	B	0.73	0.928
22	56	2.2 × 1.3	877	S	N	0.97	0.835
23	74	1.2 × 0.8	456	L	B	1.04	1.294
24	45	1.5 × 1.5	356	M	N	0.97	0.879
25	45	1.3 × 0.8	776	S	B	1.11	1.111
26	59	2.1 × 1.3	935	L	N	1.02	0.840
